# Prolonged persistence of tissue‐resident memory cells in the upper airway following SARS‐CoV‐2 infection and vaccination

**DOI:** 10.1002/cti2.70075

**Published:** 2026-01-11

**Authors:** Hyunkyung Cha, Jina Won, Soo Min Kim, Suhyun Lim, Sujin Kim, Siyeon Jin, Sung Dong Cho, Hyun Jik Kim

**Affiliations:** ^1^ Department of Otorhinolaryngology‐Head and Neck Surgery Soonchunhyang University Cheonan Hospital Cheonan Korea; ^2^ Department of Otorhinolaryngology Seoul National University College of Medicine Seoul Korea; ^3^ Department of Otorhinolaryngology Seoul National University Hospital Seoul Korea; ^4^ Sensory Organ Research Institute Seoul National University Medical Research Center Seoul Korea

**Keywords:** localised immune memory, nasopharyngeal lymphoid tissues, SARS‐CoV‐2, tissue‐resident memory T and B cells

## Abstract

**Objectives:**

Here, we characterised the diversity and persistence of immunological memory cells—particularly tissue‐resident memory T (T_RM_) and B (B_RM_) cells—in the nasopharyngeal lymphoid tissues of healthy vaccinated (HV) individuals and those who experienced SARS‐CoV‐2 breakthrough infection (BR).

**Methods:**

Nasopharynx (NP) samples were obtained using brushing from HV and BR subjects. Immune cell populations were analysed using transcriptomic profiling and flow cytometry.

**Results:**

Transcriptomic profiling revealed that the NP of SARS‐CoV‐2‐infected individuals exhibited distinctive signatures of lymphocyte‐mediated immunity, underscoring its role as a key site for viral invasion and immune activation. Effector memory CD4+ and CD8+ T (T_EM_) cells, along with non‐germinal center (GC) B cells, predominated in the NP. Although overall frequencies of memory T cells were comparable between HV and BR groups, CD4+ T_EM_ and GC B cells were significantly enriched in the NP of BR individuals at least 1 year post infection. Notably, over 80% of CD4+ T_EM_ and 40% of CD8+ T_EM_ cells were T_RM_, and more than 30% of memory B cells exhibited a B_RM_ phenotype. These populations of CD4+, CD8+ T_RM_ and B_RM_ persisted in the NP for over 2 years following SARS‐CoV‐2 infection or vaccination. In particular, CD4+ T_RM_ cells were significantly more abundant and durably maintained in the NP mucosa of BR individuals.

**Conclusion:**

Our findings identify the nasopharynx as a key site of long‐lived immunological memory, marked by persistent T_RM_ and B_RM_ cells after SARS‐CoV‐2 exposure.

## Graphical abstract

In this study, we found that both SARS‐CoV‐2 infection and vaccination elicit long‐lived tissue‐resident immune responses in the nasopharynx (NP), marked by persistent T_RM_ and B_RM_ cells. Our experiment data determine that subjects with breakthrough infections exhibit significantly enhanced frequencies and polyfunctionality of CD4^+^ T_RM_ cells, along with elevated germinal centre (GC) B‐cell responses in the NP mucosa.

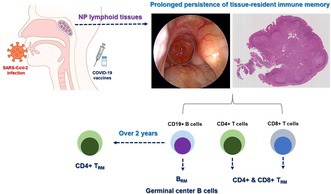

## Introduction

Pathogenic respiratory viruses elicit both humoral and cellular immune responses in humans, which are typically measured through antibody titres and T‐cell responses in peripheral blood.^1^, ^2^, ^3^, ^4^ However, little is known about the immune responses occurring in the lymphoid tissues of the upper airway—the primary sites of viral entry and replication.^5^, ^6^ Several studies have identified SARS‐CoV‐2 target sites in the upper airway, including the nasal mucosa, nasopharynx (NP) and oropharynx.^6^, ^7^, ^8^ Following SARS‐CoV‐2 infection, antiviral mechanisms in the upper airway act to limit viral replication and often prevent disease progression.^7^ Importantly, failure of these early responses is associated with progression to severe COVID‐19, indicating that impaired immunity in the upper airway may precede and contribute to systemic disease.^8^ Therefore, a better understanding of the immunological features of the upper airway may inform more effective strategies to block viral transmission and attenuate disease severity. SARS‐CoV‐2 infection induces adaptive immune responses characterised by the activation of virus‐specific T and B cells that mediate viral clearance at infection sites and limit dissemination through effector and antibody responses.^9^, ^10^ Vaccine‐induced immune memory, particularly memory T and B cells, provides more durable protection than natural infection.^11^, ^12^ Defining the breadth and function of these memory cells is essential to improve vaccine strategies against evolving SARS‐CoV‐2 variants.

While peripheral blood analysis has been informative, adaptive immune responses are also generated and maintained in various tissues, including primary infection sites and associated lymphoid organs.^4^, ^13^, ^14^, ^15^ Virus‐specific CD4+ and CD8+ memory T cells include both circulating and tissue‐resident memory T cells (T_RM_), the latter of which are non‐circulating and reside long‐term in tissues.^16^, ^17^, ^18^, ^19^ T_RM_ cells in the respiratory tract are critical for rapid and localised immune responses upon re‐exposure to SARS‐CoV‐2.^17^, ^18^

NP lymphoid tissues (adenoids) are secondary lymphoid structures with features of both lymph nodes and respiratory mucosa.^7^ We hypothesised that NP tissues are key sites for the establishment of immunological memory in the upper airway, maintaining local antigen‐specific T‐ and B‐cell responses. Recent studies have reported SARS‐CoV‐2‐specific T cells in NP lymphoid tissue, underscoring the NP's role in localised antiviral defence.^20^, ^21^, ^22^ However, the composition, distribution and long‐term persistence of tissue‐resident memory cell populations in the NP remain poorly understood.^20^

In this study, we aimed to characterise localised immune memory cell populations, particularly CD4+ and CD8+ T_RM_ and B_RM_ cells in NP lymphoid tissues. We also evaluated long‐term persistence and functional significance of T_RM_ and B_RM_ cells in the NP following SARS‐CoV‐2 infection and vaccination.

## Results

### Clinical and immunological characteristics of the NP

**Figure 1:**
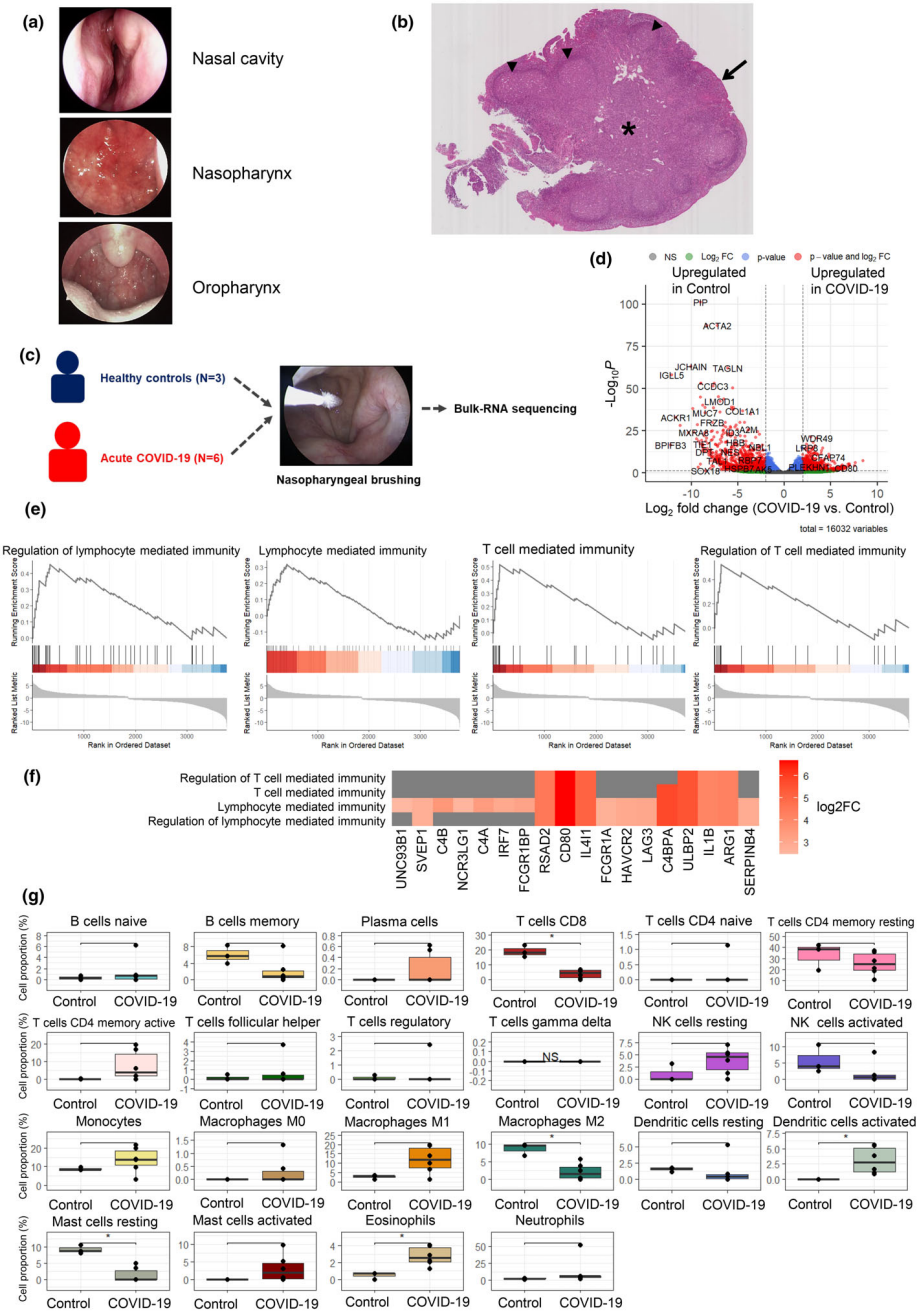
The distinctive clinical and immunologic characteristics of the NP. **(a)** Representative endoscopic findings of the nasal cavity, nasopharynx (NP), and oropharynx of three acute COVID‐19 patients (CoV2+). **(b)** A nasopharyngeal lymphoid tissue was sampled through an intranasal endoscope. The histologic structure of haematoxylin and eosin–stained NP lymphoid tissue is shown. Pseudostratified columnar epithelium (black arrow), squamous epithelium (white arrow), enlarged lymphoid follicles (black triangle), and intercellular T cell zones (*) are characteristic of NP lymphoid tissue (Scale bar: 100 μM). **(c)** Study scheme for the bulk‐RNA sequencing using NP samples from subjects with (*N* = 6) or without SARS‐CoV‐2 infection (*N* = 3). **(d)** Volcano plot comparing gene expression in the nasopharyngeal tissue of COVID‐19 patients versus the nasal mucosa of healthy controls. **(e)** Gene set enrichment analysis revealed significant enrichment of immune‐related GO terms, including regulation of lymphocyte‐mediated immunity, lymphocyte‐mediated immunity itself, T‐cell‐mediated immunity, and regulation of T‐cell‐mediated immunity. **(f)** A term‐gene heatmap illustrating the log_2_ fold change of core enrichment genes associated with significantly enriched GO terms from GSEA. **(g)** Immune cell proportions comparing healthy nasal mucosa and COVID‐19‐infected nasopharyngeal tissue, estimated using CIBERSORTx. The analysis revealed a significant decrease in CD8^+^ T cells (*P* = 0.024) and a trend toward increased CD4^+^ memory activated T cells (*P* = 0.120).

To evaluate localised immune responses in the NP following SARS‐CoV‐2 infection or vaccination, we first examined the clinical, histological and immunological features of the NP in acute COVID‐19 patients (CoV2+). Three patients with confirmed SARS‐CoV‐2 infection presented with fever, chills, myalgia, sore throat and anosmia persisting for more than 3 days. Endoscopic examination of the nasal cavity (NC), NP and oropharynx (OP) revealed that while the NC and OP mucosa appeared normal, the NP showed marked signs of inflammation, including mucosal erosion, erythema and redness (Figure 1a).

To compare viral entry potential, we assessed ACE2 expression in NP versus NC mucosa from 12 healthy individuals. Quantitative PCR revealed significantly higher ACE2 mRNA levels in the NP (Supplementary figure 1). Histological analysis of NP mucosal tissue from a healthy donor confirmed the presence of pseudostratified columnar and squamous epithelium, along with prominent lymphoid follicles, germinal centres and interfollicular T‐cell zones—features distinct from NC mucosa of our previous study (5) (Figure 1b).

We next profiled NP transcriptomes from CoV2+ subjects (*n* = 6) and SARS‐CoV‐2–negative controls (*n* = 3) using bulk RNA sequencing. We collected demographic data, including sex, age, number of vaccine doses and the period from last vaccination (Table 1). Control samples were from three subjects who underwent NP brushings prior to the 2020 COVID‐19 pandemic infection, and RNA was extracted and used for bulk RNA seq. A total of 618 genes were upregulated and 920 downregulated in CoV2+ NPs (log_2_FC ≥ 2, adjusted *P* < 0.05; Figure 1c and d). Gene set enrichment analysis revealed significant upregulation of immune‐related pathways, including regulation of lymphocyte‐mediated immunity and T‐cell–mediated responses (Figure 1e). It shows high expression of CD80—an immune checkpoint protein primarily expressed on antigen‐presenting cells, including B cells and T cells—as well as cytokines such as IL4I1 and IL1B (Figure 1f).

**Table 1:** Baseline characteristics of recruited subjects, related to Figure 1

	Healthy control	Acute COVID‐19
Number of patients (*n*)	3	6
Mean age (years)	42.1	40.9
Sex (M:F)	2:1	3:3
Number of vaccinations	0	2.54
Time from last vaccination (months)		12.2
< 12 months (patients' number)		4
12 ≤–< 24 months (patients' number)		1
≥ 24 months (patients' number)		1

Immune cell deconvolution using CIBERSORTx estimated relative immune cell fractions in the nasopharynx during acute SARS‐CoV‐2 infection. CD8^+^ T‐cell proportions were significantly reduced in CoV2+ samples (*P* = 0.024), whereas activated dendritic cells (*P* = 0.026) and eosinophils (*P* = 0.024) were significantly increased. We also observed a trend towards reduced resting CD4^+^ memory T cells, which may partially reflect a shift from a resting to an activated state, consistent with the increasing trend in activated CD4^+^ memory T cells. Similarly, decreased memory B‐cell proportions during acute infection may occur alongside an increase in plasma cells, reflecting activation and differentiation. Monocytes, M1 macrophages, activated mast cells, and neutrophils also showed trends towards increased proportions (Figure 1g). Collectively, these findings support the nasopharynx as a primary site of upper airway SARS‐CoV‐2 infection and exhibit distinct histologic features consistent with immune activation. We planned further analyses to examine how immune memory cell populations vary with SARS‐CoV‐2 infection and vaccination status.

### The composition of immune memory cells in healthy NP mucosa

**Figure 2:**
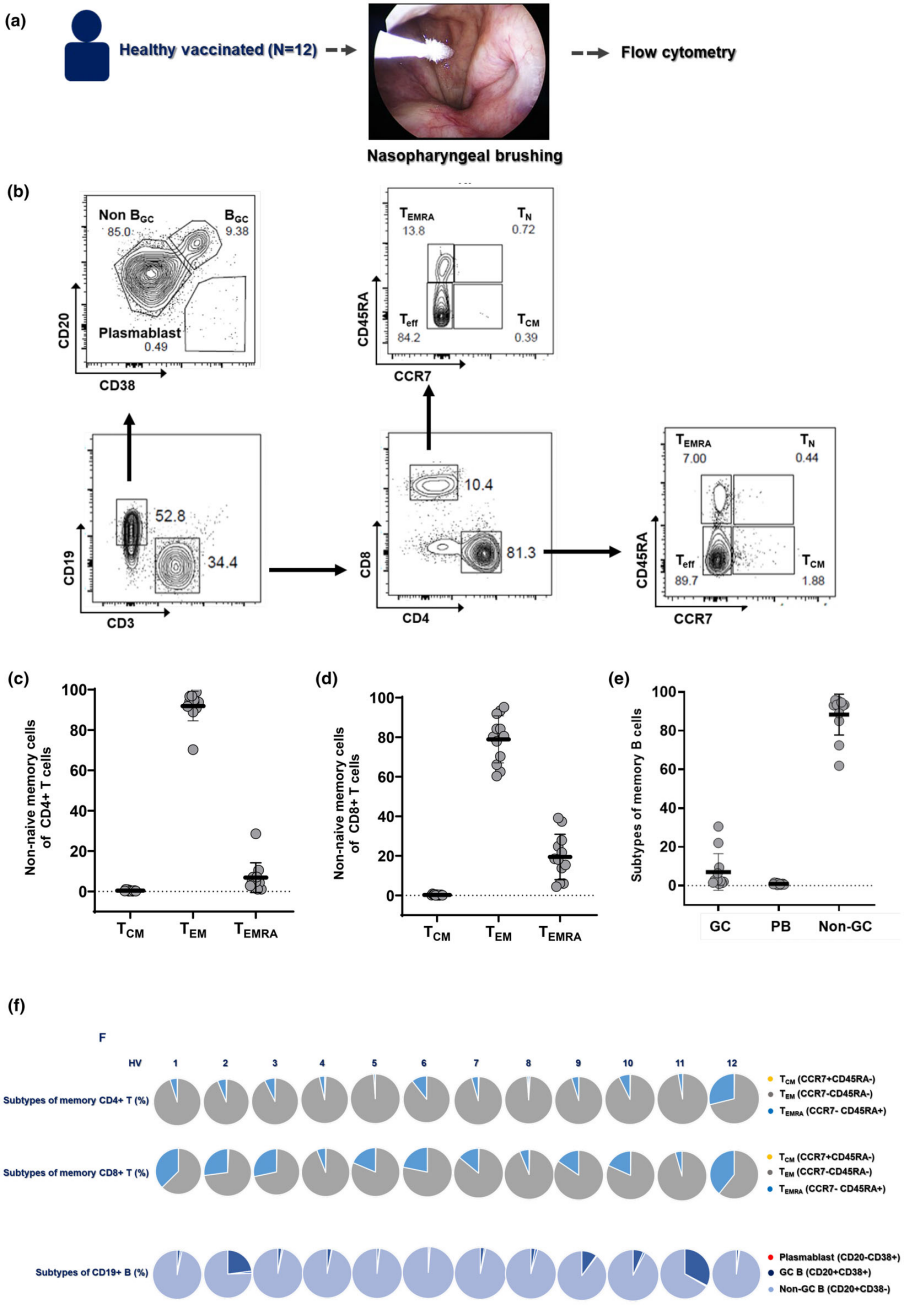
Distinct immune cell proportions in the NP mucosa of healthy vaccinated. **(a)** Study scheme for the flow cytometry using NP brushing samples (*N* = 12). **(b)** Representative flow cytometry of CD4+, CD8+ T and CD19+ B cells and subtypes of memory T and B cells in NP mucosa. Differences in immune memory cell frequencies such as **(c)** CD4+ T cells, **(d)** CD8+ T cells, and **(e)** CD19+ B cells in the NP brushings of healthy subjects. **(f)** Individual data of memory CD4+, CD8+ and CD19+ B cells subtypes.

To characterise the baseline immunological landscape, we analysed the adaptive immune cells in NP of 12 healthy vaccinated subjects (HV) by flow cytometry. The demographic data, including sex, age, number of vaccine doses, types of vaccines and the period from last vaccination, are shown in Table 2. CD4^+^ and CD8^+^ memory T‐cell subsets were defined using CCR7 and CD45RA, and B‐cell subsets using CD20 and CD38 (Figure 2a and b). Both CD4^+^ and CD8^+^ T cells predominantly exhibited an effector memory phenotype (T_EM_; CCR7^−^CD45RA^−^), while 6–20% were terminally differentiated effector cells (T_EMRA_; CCR7^−^CD45RA^+^) (Figure 2c and d). Most CD19^+^ B cells exhibited a non‐germinal center (non‐GC) phenotype (CD20^+^CD38^−^), with select individuals showing elevated frequencies of GC B cells (CD20^+^CD38^+^) (Figure 2e).

**Table 2:** Baseline characteristics of recruited subjects, related to Figure 2

	HV
Number of patients (*n*)	12
Mean age (years)	39.5
Sex (M:F)	7:5
Number of vaccinations	3.13
Types of vaccines	
AstraZeneca	0
Pfizer	7
Moderna	4
Janssen + Pfizer	0
AstraZeneca + Pfizer	1
AstraZeneca + Moderna	0
Time from last vaccination (months)	17.7
< 12 months (patients' number)	1
12 ≤–< 24 months (patients' number)	7
≥ 24 months (patients' number)	4

The ratio of CD4+ and CD8+ memory T‐cell and B‐cell subtypes was analysed for each donor based on flow cytometry data. When the total number of CD4+ T cells was set as 100%, donor‐specific analysis revealed that CD4^+^ T_EM_ cells were dominant across all subjects, with one HV donor (#12) exhibiting a higher T_EMRA_ frequency (26.8%). Among CD8+ T cells, CD8^+^ T_EM_ cells were also consistently enriched, though CD8^+^ T_EMRA_ proportions were elevated (> 10%) in several subjects (#1, 2, 3, 5, 6, 7, 9, 10, 12). The proportion of each B‐cell subtype distributed in the NP among CD8+ T cells was converted to a percentage of total B cells. The result revealed that non‐GC B cells predominated in most samples, whereas GC B‐cell enrichment was observed in subjects #2 and #11 (Figure 2f). These data indicate that the healthy NP is enriched in CD4^+^ T_EM_, CD8^+^ T_EM_ and non‐GC B cells, with inter‐individual variability in CD8^+^ T_EMRA_ and GC B‐cell populations.

### The profiles of immune memory cells in the NP mucosa depending on SARS‐CoV‐2 exposure and vaccination status

**Figure 3:**
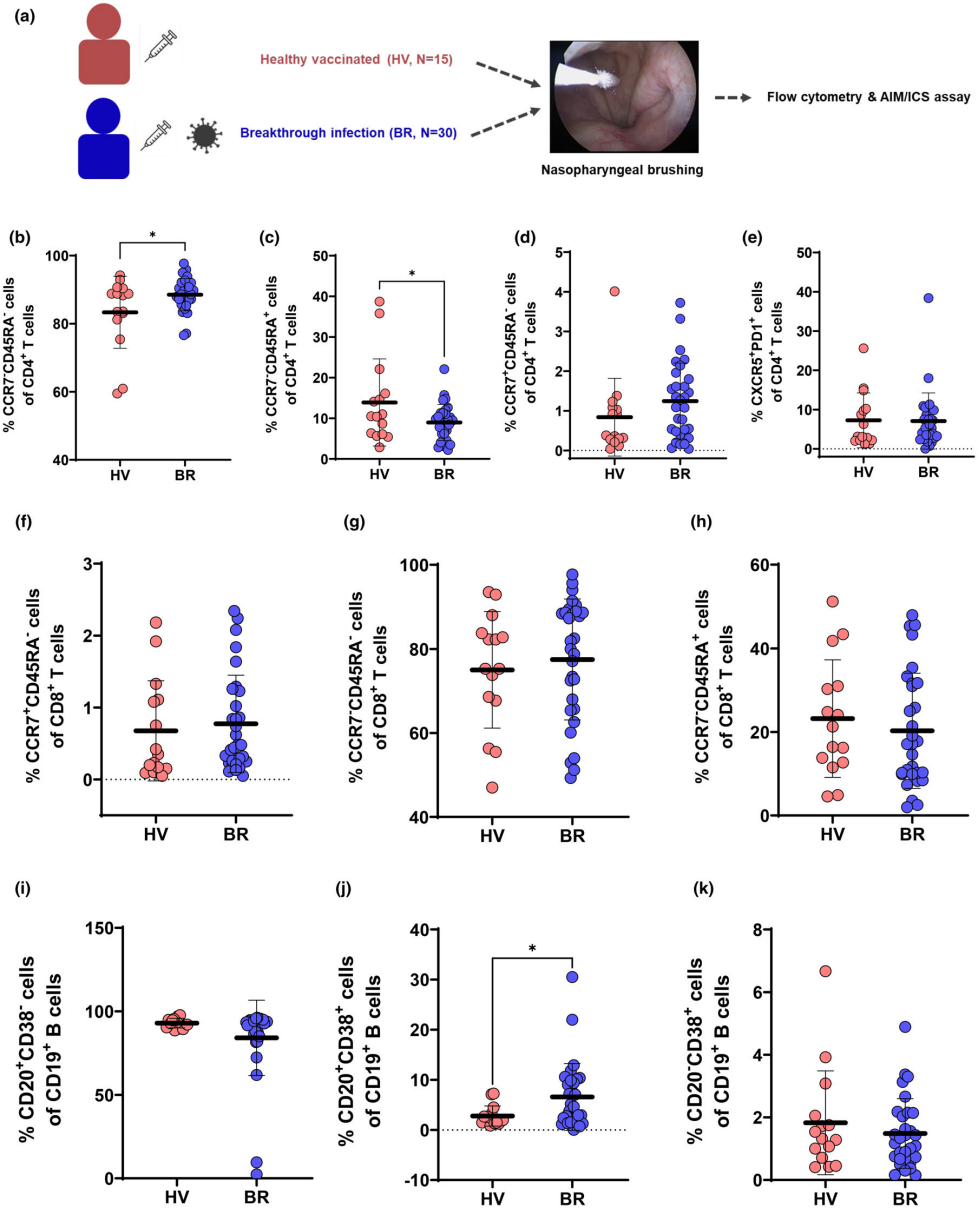
The frequencies of memory T and B cells in the NP mucosa of HV and BR subjects. **(a)** Study scheme for the flow cytometry and AIM/ICS assay using NP mucosal samples from healthy vaccinated (HV, *N* = 15) and breakthrough (BR, *N* = 30) subjects. Differences in subtype profiles of NP CD4+ memory T cells according to expression of CCR7 and CD45RA in the NP mucosa. Comparisons of **(b)** percentage of CD4+ T_EM_, **(c)** percentage of CD4+ T_EMRA_, **(d)** percentage of CD4+ T_CM_, **(e)** percentage of Tfh (follicular helper T cells), **(f)** percentage of CD8+ T_EM_, **(g)** percentage of CD8+ T_EMRA_, **(h)** percentage of CD8+ T_CM_, **(i)** percentage of CD19+ non‐germinal center (GC) B cells (CD20+CD38−), **(j)** percentage of CD19+ GC B cells (CD20+CD38+), and **(k)** percentage of CD19+ plasmablast (CD20−CD38+) in NP mucosa (mean ± standard deviation: nonparametric *t* test, *P < 0.05).

We profiled immune memory cell populations in NP brushings from 45 individuals: vaccinated SARS‐CoV‐2–naïve (HV, *n* = 15) and vaccinated with breakthrough infection (BR, *n* = 30) (Figure 3a, Table 3). Subjects included in this study received 2 or 3 doses of COVID‐19 vaccines and were diagnosed with COVID‐19 at least once (BR). HV subjects underwent NP brushings an average of 23.9 months after the last vaccination, and BR on average 27.1 months following their last vaccination (Table 3). We analysed the period between vaccination and COVID‐19 diagnosis in 30 BR subjects (Supplementary tables 1 and 2). The demographic data revealed that BR subjects underwent NP brushing at approximately 8 months after COVID‐19 confirmation and the mean period between last vaccination and COVID‐19 was 18.7 months.

**Table 3:** Baseline characteristics of recruited subjects, related to Figure 3

	HV	BR
Number of patients (*n*)	15	30
Mean age (years)	37.4	46.1
Sex (M:F)	9:6	17:13
Number of vaccinations	2.82	2.86
Types of vaccines		
AstraZeneca	0	1
Pfizer	8	12
Moderna	4	7
Janssen + Pfizer	0	2
AstraZeneca + Pfizer	3	7
AstraZeneca + Moderna	0	1
Time from last vaccination (months)	20.8	27.1
< 12 months (patients' number)	2	1
12 ≤–< 24 months (patients' number)	8	9
≥ 24 months (patients' number)	5	20

As a next step, we compared the population of adaptive immune cells in the NP of HV and BR subjects. The results of flow cytometry showed that no significant differences in total CD4^+^, CD8^+^ or CD19^+^ cell frequencies were observed between HV and BR groups (Supplementary figure 2a–d). Contrasting total frequencies of T and B cells, phenotyping of memory T cells revealed significantly higher frequencies of CD4^+^ T_EM_ and lower CD4^+^ T_EMRA_ in BR subjects, while central memory (T_CM_, CCR7+CD45RA−) CD4^+^ T cells and CD4+ follicular helper T (Tfh, CXCR5+PD1+) cells were comparable between groups (Figure 3b–e). CD8^+^ T_EM_, T_CM_ and T_EMRA_ subsets did not differ significantly (Figure 3f–h). The analysis of CD19+ B cells revealed a significantly higher proportion of GC B cells in the NP of BR donors, while non‐GC and plasmablast (CD28‐CD38+) populations were not comparable between HV and BR subjects (Figure 3i–k).

**Figure 4:**
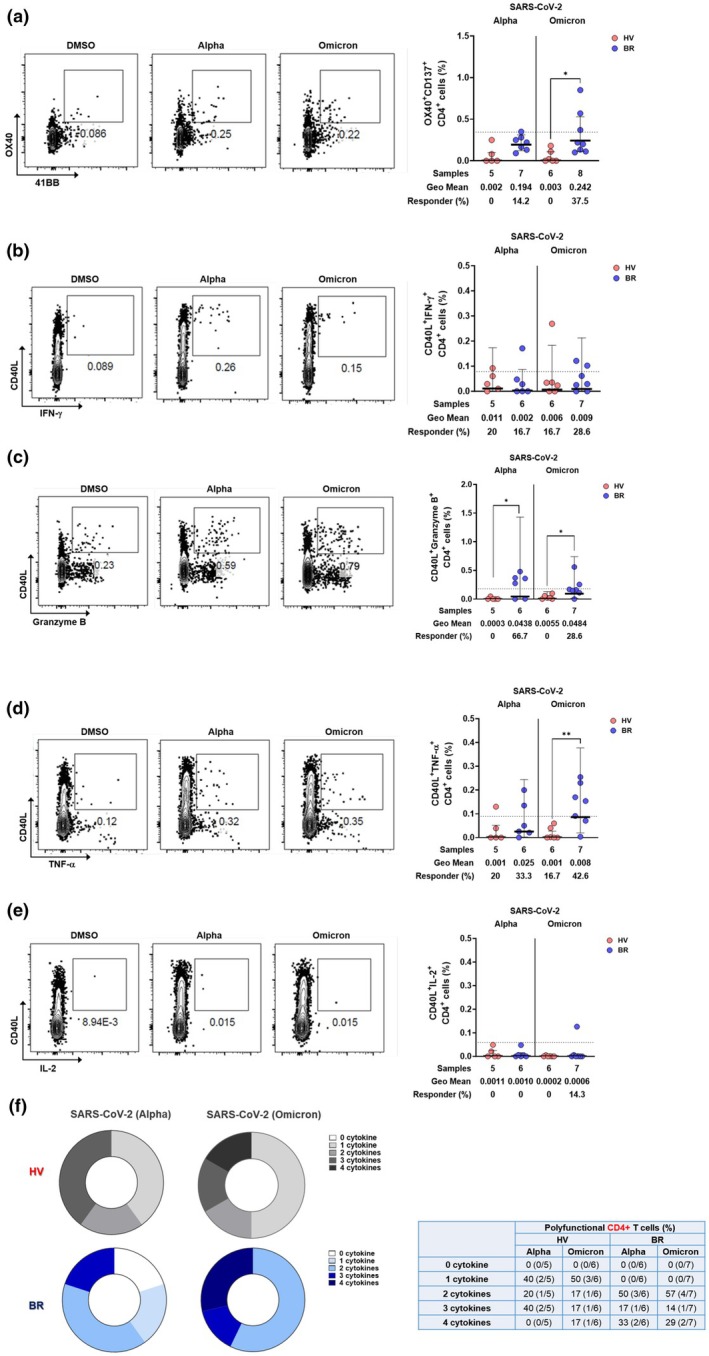
SARS‐CoV‐2‐specific memory CD4+ T cells in the NP mucosa of healthy vaccinated and breakthrough donors. Differences in subtype profiles of SARS‐CoV‐2‐specific CD4+ memory T cells and ratios in the NPs of the healthy vaccinated (HV, *N* = 15) and breakthrough (BR, *N* = 30) subjects by AIM and ICS assay. **(a)** Representative flow cytometry plots of SARS‐CoV‐2‐specific CD4+ T cells (OX40+ 41BB+) in HV and BR donors and percentage of background‐subtracted spike‐specific CD4+ T cells (surface OX40+41BB+, as percentage of CD4+ T cells) by AIM assay following 24‐h stimulation of NP immune cells with alpha and omicron spike megapools (MPs). Representative flow cytometry plots of SARS‐CoV‐2‐specific IFN‐γ+CD4+ T cells (CD40L+IFN‐γ+) in the NP of HV and BR donors and percentage of background‐subtracted spike‐specific CD4+ T cells (surface CD40L+ intracellular IFN‐Υ positive, as percentage of CD4+ T cells) by hybrid AIM+ICS following 24‐h stimulation with SARS‐CoV‐2 alpha and omicron spike MPs for IFN‐γ **(b)**. Granzyme B **(c)**, TNF‐α **(d)**, and IL‐2 **(e)**. **(f)** Donut charts representing the proportion of antigen‐specific CD4+ T cells producing 0–4 cytokines in the NP of HV and BR donors (nonparametric *t* test, *P < 0.05, **P < 0.01).

To quantify antigen‐specific responses, CD4^+^ T cells were stimulated with spike peptide megapools (alpha and omicron variants) and analysed by activation‐induced marker (AIM) assays. Omicron‐specific AIM^+^CD4^+^ T cells were significantly enriched in BR donors, with 14.2% and 37.5% responding to alpha and omicron spike peptides, respectively (Figure 4a). Functional profiling via intracellular cytokine staining (ICS) showed that GzmB^+^ and TNF^+^ CD4^+^ T‐cell responses were significantly elevated in BR donors following stimulation with both variants, while IFN‐γ and IL‐2 responses were not (Figure 4b–d). Notably, SARS‐CoV‐2–specific CD4^+^ T cells in BR donors exhibited greater polyfunctionality, with a higher proportion producing ≥ 2 cytokines (Figure 4e). In contrast to CD4+ T cells, SARS‐CoV‐2–specific and polyfunctional CD8^+^ T cells were not observed in the NP of HV and BR donors (Supplementary figure 3). The current findings indicate that localised immune memory T and B cells tended to be altered in the NP depending on SARS‐CoV‐2 infection and vaccination status. The SARS‐CoV‐2 infection following vaccination enhances CD4+ T_EM_ cell abundance, cytokine functionalities of CD4+ T cells and GC responses in the NP mucosa. The population of AIM^+^CD4^+^ T cells and polyfunctionality were significantly lower in the NP of HV subjects. We presumed that subjects who received only the COVID‐19 vaccine had shorter durations of virus‐specific CD4+ T cells in the NP compared to BR donors.

### Long‐term persistence of T_RM_ and B_RM_ cells in the NP

**Figure 5:**
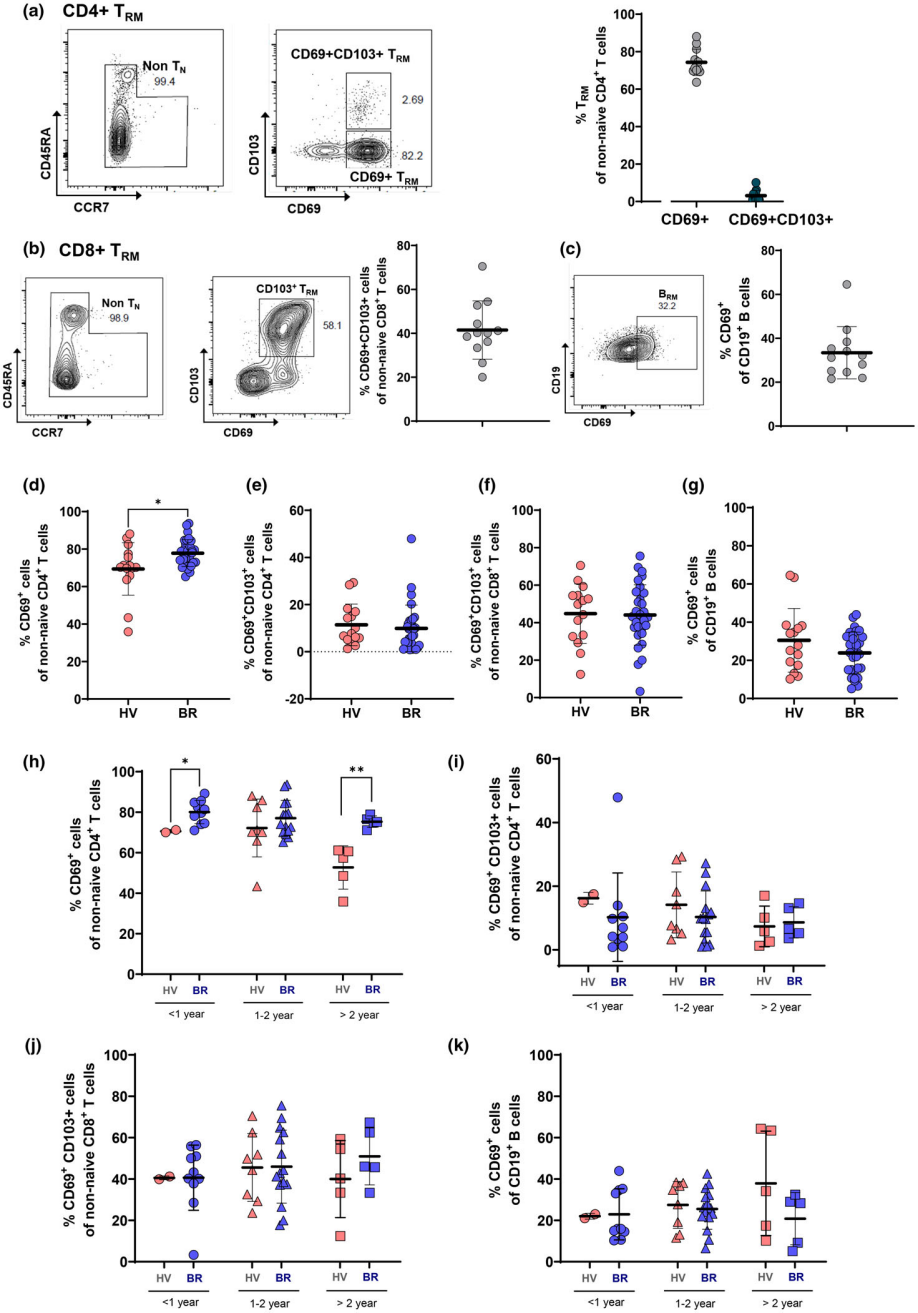
The frequencies of tissue‐resident memory CD4+, CD8+ T and CD19+ B cells in the NPs of HV and BR subjects. **(a)** Representative flow cytometry plots for CD4+ T_RM_ (CD69+ and CD69+C103+) and the frequencies of CD69+CD4+ T cells and CD69+CD103+CD4+ T cells as percentage of CD4+ T cells. **(b)** Representative flow cytometry plots for CD8+ T_RM_ (CD69+C103+ T cells) and the frequency of CD69+CD103+CD8+ T cells as percentage of CD8+ T cells. **(c)** Representative flow cytometry plots for CD19+ B_RM_ (CD69+C19+ B cells) and the frequency of CD69+CD19+ B cells as percentage of CD19+ B cells. The results of **(a)**, **(b)**, and **(c)** were from NP brushings of 12 HV as used in Figure 2. **(d)** Difference in the frequencies of CD69+CD4+ T cells, **(e)** CD69+CD103+CD4+ T cells, **(f)** CD69+CD103+CD8+ T cells, and **(g)** CD69 + CD19+ B cells in the NP mucosa of HV and BR subjects (mean ± standard deviation: nonparametric *t* test, *P < 0.05). **(h)** Difference in the frequencies of CD69+CD4+ T cells, **(i)** CD69+CD103+CD4+ T cells, **(j)** CD69+CD103+CD8+ T cells, and **(k)** CD69+CD19+ B cells in the NP mucosa of HV (*N* = 15) and BR (*N* = 30) subjects according to the period of last SARS‐CoV‐2 infection and vaccination (mean ± standard deviation: nonparametric t test, *P < 0.05, **P < 0.01).

We next assessed the frequency and persistence of tissue‐resident memory T (T_RM_) and B (B_RM_) cells in the NP of 12 HV (Table 2). Flow cytometry results revealed that CD4^+^ T_RM_ cells were defined as CD69^+^ non‐naïve CD4^+^ T cells, with CD103 co‐expression rarely observed (Figure 5a). CD4^+^ T_RM_ cells composed of 92.5% of non‐naïve CD4^+^ T cells (mean: 72.3%). CD8^+^ T_RM_ cells (CD69^+^CD103^+^ among non‐naive CD8^+^ T cells) were present at a mean of 41.7% (Figure 5b). B_RM_ cells, defined as CD69^+^ among CD19^+^ B cells, had a mean frequency of 37.6% (Figure 5c). Then, we profiled the populations of T_RM_ and B_RM_ in NP brushings from 45 individuals (HV, *n* = 15 and BR, *n* = 30, Table 3) with the same gating strategy. Comparative analysis showed that CD4^+^ T_RM_ cells were significantly abundant in the NP mucosa of BR than HV donors (Figure 5d), whereas CD8^+^ T_RM_ and B_RM_ frequencies were similar (Figure 5e–g).

To evaluate cellular persistence, we stratified donors into three groups based on time since last vaccination or infection: < 1 year, 1–2 years and > 2 years. CD69^+^CD4^+^ T_RM_ frequencies were significantly higher in BR donors at both < 1 year and > 2 years post exposure, while CD69^+^CD103^+^CD4^+^ T_RM_ proportions remained unchanged (Figure 5h and i). The proportions of CD8+ T_RM_ and B_RM_ did not differ between the two groups according to last vaccination or SARS‐CoV‐2 infection, but CD8^+^ T_RM_ and B_RM_ cell frequencies remained stable over time in both groups (Figure 5j and k).

These findings indicate that T_RM_ and B_RM_ cells persist in the NP mucosa for over 2 years following SARS‐CoV‐2 exposure and vaccination, with significantly enhanced longevity of CD4^+^ T_RM_ cells in individuals with breakthrough infection.

## Discussion

In this study, we demonstrate that nasopharyngeal (NP) lymphoid tissues serve as a distinct immunological niche that supports the generation and long‐term maintenance of memory T and B cells following SARS‐CoV‐2 infection and vaccination. We found that CD4^+^, CD8^+^ T_RM_ cells and B_RM_ cells persisted in the NP for more than 2 years after the last antigen exposure. Notably, CD4^+^ T_RM_ and SARS‐CoV‐2–specific CD4^+^ T cells were significantly more abundant in the NP of subjects with BR infections compared to HV subjects, even over 1 year after infection. These findings underscore the NP as a key site of localised immunological memory and highlight the role of infection history in shaping tissue‐resident antiviral immunity.

As the primary interface for inhaled pathogens, the NP represents a critical site for the early containment of respiratory viruses such as SARS‐CoV‐2.^23^, ^24^, ^25^ The NP lymphoid tissue, with its dual features of respiratory mucosa and secondary lymphoid architecture, provides a specialised microenvironment for the generation of adaptive immune responses.^20^, ^26^, ^27^, ^28^ While antigen‐specific memory T and B cells are detectable in peripheral blood for up to 8 months after vaccination, little is known about the long‐term persistence and tissue residency of these cells in the upper airway in response to SARS‐CoV‐2 exposure and vaccination status.^2^ Our findings reveal a distinct immune landscape in the NP, with dominant populations of CD4^+^ T_EM_, CD8^+^ T_EM_, and non‐GC B cells, and a relative paucity of circulating memory T cells such as T_CM_ and T_EMRA_. This suggests that immunological memory T and B cells would also be detectable in the NP lymphoid tissue of individuals who received COVID‐19 vaccines by intramuscular injection or who were diagnosed with COVID‐19 after vaccination and the NP supports a memory compartment distinct from that of the systemic immune system.

Importantly, both CD4^+^ and CD8^+^ memory T cells, as well as B_RM_ cells, were detectable in the NP long after intramuscular COVID‐19 vaccination, indicating dissemination of vaccine‐induced immune memory to mucosal tissues. Subjects with breakthrough infections exhibited significantly higher frequencies of CD4^+^ T_EM_ and GC B cells, as well as enhanced SARS‐CoV‐2–specific CD4^+^ T‐cell responses, including polyfunctionality and cytokine production. These data support the hypothesis that mucosal re‐exposure via natural infection enhances the breadth and functionality of tissue‐resident memory responses, in contrast to vaccination alone.^1^, ^29^

We assumed that both T_RM_ and B_RM_ cells might be involved in the initial activation of mucosal immunity in the NP mucosa, the target tissue for SARS‐CoV‐2 infection and their persistence in the NP following SARS‐CoV‐2 exposure is likely to contribute to rapid viral recognition and containment upon re‐infection. Previous studies have identified SARS‐CoV‐2–specific T_RM_ cells in lymph nodes, lungs and other non‐lymphoid tissues.^30^, ^31^, ^32^ T_RM_ cells play a pivotal role in controlling secondary infections through localised, antigen‐specific effector responses.^4^ Moreover, B_RM_, plasma cells and GC B cells enriched in upper airway tissues have been shown to include IgA^+^ subsets and display compartmentalised responses distinct from those in circulation.^33^ Evidence of T_RM_ or B_RM_ persistence in the upper airway, which may represent a reservoir of long‐lived mucosal immunity in the human upper airway, was demonstrated in the present study. Recent studies of mice treated with different vaccines for SARS‐CoV‐2 mutations eliciting SARS‐CoV‐2‐specific T_RM_ in the upper airway further confirmed the importance of tissue‐resident mucosal immunity in SARS‐CoV‐2 control.^34^, ^35^ Both T_RM_ and B_RM_ cells that quickly recognise virus‐producing infected cells in the respiratory tract can play a critical role in rapidly containing and eliminating SARS‐CoV‐2 infection.^36^, ^37^, ^38^ We demonstrated that both CD8+ and CD4+ T_RM_ cells are primarily present in the NP among non‐naïve T cells and that the total proportions of these memory populations were stably maintained in the NP mucosa in HV and BR individuals. Our data showed that more than 60% of the non‐naive CD4+ were CD4+ T_RM_ and more than 40% of CD8+ T cells were CD8+ T_RM_. In particular, the proportion of CD4+ T_RM_ was significantly higher in the NP of BR donors and persisted this way for longer than 2 years from the last SARS‐CoV‐2 infection. Our findings extend this concept to the NP mucosa, demonstrating stable T_RM_ and B_RM_ populations, particularly CD4^+^ T_RM_ cells in both HV and BR groups.

Interestingly, while CD8^+^ T_RM_ cells were also maintained for over 2 years, their frequencies did not differ significantly between HV and BR individuals, suggesting differential mechanisms of maintenance or recall between T‐cell subsets. B_RM_ cells composed of over 30% of CD19^+^ B cells in the NP and remained stable for more than 2 years post exposure. These data suggest that the NP serves as a long‐term reservoir for tissue‐resident memory T and B cells, which may be strategically positioned to mediate early control of reinfection.

We hypothesise that the accumulation and persistence of T_RM_ and B_RM_ cells in the NP reflect site‐specific imprinting of immune memory, potentially providing durable mucosal protection. The elevated presence of SARS‐CoV‐2–specific CD4^+^ T_RM_ cells following breakthrough infection indicates that natural infection boosts tissue‐localised memory, which may be less robust following systemic vaccination alone. The dominance of CD4^+^ T cells in the NP mucosa, together with their heightened functionality in BR individuals, supports their role in mucosal antiviral immunity. Moreover, our data suggest that T_RM_ and B_RM_ cells could be targeted by mucosal vaccine strategies aimed at enhancing site‐specific immunity.

This study has several limitations. We did not include data on other respiratory viruses such as influenza or rhinovirus, which may affect memory cell dynamics. Longitudinal sampling was not available, precluding analysis of temporal dynamics within individuals. Additionally, peripheral blood comparisons were not performed, and unexposed, unvaccinated controls were not available because of the timing of the pandemic in Korea.

Despite these limitations, our results provide key insights into the persistence and composition of immune memory cells in the NP. Both SARS‐CoV‐2 infection and vaccination elicited durable tissue‐resident immune responses, particularly among the CD4^+^ T_RM_ population. These responses were functionally robust, with enhanced antigen specificity and cytokine production of CD4+ T cells in subjects with BR infection. Together, our findings highlight the NP mucosa as an immunologically privileged site that harbours long‐lived T_RM_ and B_RM_ cells, contributing to localised antiviral memory and potential protection against future SARS‐CoV‐2 variants.

## Methods

### Study participants

NP lymphoid tissues were collected from laboratory‐confirmed COVID‐19 patients who were hospitalised between May 2022 and June 2022 during the Omicron era at Seoul National University Hospital (*N* = 6). The NP brushings from healthy controls were obtained during nasal surgery, which was performed before the COVID‐19 pandemic (*n* = 3) (Table 1). Twelve HV subjects were enrolled in this study for Figures 2 and 4a–c, 24 males (mean body mass index 21.5 kg/m^2^) and 21 females (mean body mass index 22.6 kg/m^2^). Their mean age was 35.2 years, and they were all referred to the Department of Otorhinolaryngology at Seoul National University Hospital (Seoul, Korea) between January 2024 and November 2024, primarily for nasal surgery. Forty‐five HV or BR subjects (Figures 3 and 4d–k) were enrolled in this study, 24 males (mean body mass index 21.5 kg/m^2^) and 21 females (mean body mass index 22.6 kg/m^2^). Their mean age was 35.2 years, and they were also referred between January 2024 and November 2024, primarily for nasal surgery. Intranasal endoscopy, computed tomography of the paranasal sinus, and a skin allergy test were performed before sampling. None of the subjects showed signs of acute infection including COVID‐19, and all showed negative results in the allergy test. Subjects who were pregnant or a smoker, had diseases or medication histories related to asthma or had any other chronic diseases (atherosclerosis, hypertension, arrhythmia, congestive heart failure, diabetes mellitus, osteoporosis, hepatitis, cancer, or autoimmune or neurological disease) were excluded. This study was performed according to the guidelines of the Helsinki Declaration and was approved by the Institutional Review Board of Seoul National University College of Medicine, Seoul, Korea (IRB No. 2305‐149‐1434). Written informed consent was obtained from all participants before sample collection. All subjects included in this study had received at least two doses of a vaccine against COVID‐19. The subjects were classified into two groups according to SARS‐CoV‐2 infection history: (1) healthy vaccinated donors (HV: vaccinated donors with no history of SARS‐CoV‐2 infection, *N* = 27), and (2) vaccinated donors with a history of SARS‐CoV‐2 infection (breakthrough cases, BR, *N* = 30). SARS‐CoV‐2 infection was confirmed by SARS‐CoV‐2 polymerase chain reaction during the omicron era in Korea.

### Sample collection and processing

NPs were accurately examined using a rigid 0‐degree intranasal endoscope to obtain high‐quality lymphoid tissues, and NP brushing was performed by an otorhinolaryngologist (HJ Kim) (Supplementary video 1). A flocked brush (Vansco, Seoul, Korea) was gently inserted into one nostril along the floor of the nasal cavity into the posterior NP (8–9 cm maximum insertion distance). Each brush sample was collected and placed in an individual tube containing RPMI (Gibco) supplemented with 10% fetal bovine serum. After collection, the brushed samples were vortexed briefly in a capped collection tube to release the cells for flow cytometry. Additional medium was used to rinse adherent cells from the brush into a 40 μm mesh filter over a 50 mL conical tube. The filtered cell suspension was centrifuged at 500 rcf for 7 min at 4°C, and then the brushed cells were processed for downstream applications.

### Flow cytometry

Isolated NP cells were stained for surface and intracellular markers (Supplementary figure 4). The antibodies and clones used are described in Supplementary table 3. Briefly, cells were washed with fluorescence‐activated cell sorting (FACS) buffer (PBS with 3% heat‐inactivated fetal bovine serum) and resuspended with a surface staining antibody cocktail for 30 min at 4°C. The surface‐stained cells were fixed and permeabilised using a Cytofix/Cytoperm kit (BD Biosciences) for 20 min at 4°C. Subsequently, the cells were resuspended with an intracellular staining antibody cocktail for 30 min at 4°C. Flow cytometry data were collected using a five‐laser FACSymphony A3 flow cytometer (BD Biosciences) and analysed using the FlowJo V 10.7 software. Dead cells were stained using a LIVE/DEAD fixable blue stain kit (Thermo Fisher Scientific) in PBS at room temperature for 15 min.

### RNA sequencing data

Publicly available RNA sequencing data were obtained from the Gene Expression Omnibus (GEO) under the accession number GSE239595. This dataset, previously published,^7^ includes transcriptomic profiles from NP samples of uninfected control individuals and those with active SARS‐CoV‐2 infection. These data were retrieved and included in our analysis.

### Differential expression and enrichment analysis

QuantSeq 3' mRNA‐Seq reads were aligned to the genome or transcriptome using Bowtie2,^39^ with indices built from either the genome assembly or representative transcripts. The resulting alignment files were used for transcript assembly, abundance estimation and differential gene expression analysis. Differentially expressed genes (DEGs) were identified using Bedtools^40^ based on read coverage, considering both uniquely and multiply mapped reads. Read counts were normalised using the median‐of‐ratios method and regularised log transformation with the DESeq2 package (v 3.2).^41^ DEGs were defined as genes with an adjusted *P*‐value < 0.05 and absolute log_2_ fold change ≥ 2. Immune cell composition was estimated with CIBERSORTx using the LM22 signature matrix, which includes 547 genes representing 22 immune cell types.^42^ TPM‐normalised raw read counts were used, with batch correction and 1000 permutations applied. Gene set enrichment analysis of upregulated DEGs was conducted using fgsea with Gene Ontology Biological Process (GO‐BP) terms. Gene set sizes were restricted with minGSSize = 10 and maxGSSize = 500. Enriched pathways were identified based on adjusted *P*‐values < 0.05.^43^

### Hybrid AIM + ICS

Activation‐induced marker (AIM) and intracellular cytokine staining (ICS) assays were used to measure T‐cell responses. For the AIM assay, isolated NP cells (1 × 10^6^) were blocked with 0.5 μg/mL anti‐human CD40 mAb (clone HB14, Miltenyi Biotec) in HR5 medium (RPMI 1640 with 5% human AB serum (Sigma), 1% GlutaMax (Thermo Fisher Scientific), and 1% penicillin and streptomycin (Thermo Fisher Scientific)) in a round‐bottom 96‐well plate for 15 min at 37°C in an incubator with 5% CO_2_. The cells were then stimulated with 1 μg/mL of SARS‐CoV‐2 spike alpha and omicron peptide pools (Genscript, Piscataway, NJ, USA) in HR5 medium for 24 h at 37°C. An equivalent amount of DMSO in HR5 medium was used as a negative control, and 1 μg/mL staphylococcal enterotoxin B (SEB; Toxin Technologies, Sarasota, Florida) served as the positive control. After incubation, the stimulated cells were treated with 0.25 μL/well of GolgiPlug (BD Biosciences), 0.25 μL/well of GolgiStop (BD Biosciences) and AIMs for 4 h at 37°C. The cells were subsequently washed and resuspended in PBS containing Live/Dead Fixable Blue at a 1:1000 dilution (Thermo Fisher Scientific) and 5 μL of human Fc block per sample (BD Biosciences) for 15 min at room temperature. Following this, the cells were washed with FACS buffer and stained with a surface antibody cocktail for 30 min at 4°C. For ICS, cells were fixed and permeabilised using a Cytofix/Cytoperm kit (BD Biosciences) following the manufacturer's instructions and then stained with a cytokine antibody cocktail for 30 min at 4°C. The ICS‐positive controls for these experiments were 0.05 μg/mL PMA (Sigma) and 0.25 μg/mL ionomycin (Sigma). AIM^+^ cells were gated based on spike peptide pool‐stimulated responses compared to DMSO‐stimulated responses. Specifically, AIM^+^CD4^+^ cells were defined as OX40^+^CD137(41‐BB)^+^ cells, and AIM^+^CD8^+^ cells were defined as CD69^+^CD137^+^ cells. For ICS, SARS‐CoV‐2 spike‐specific CD4^+^ T cells were gated using CD40L, and CD8^+^ T cells were gated using CD69, for cytokines including IFN‐γ, Granzyme B, TNF‐α, and IL‐2.

The Stimulation Index (SI) was calculated as fold change by dividing the frequency of spike peptide pool‐stimulated AIM^+^ cells by the frequency of DMSO‐stimulated AIM^+^ cells. A positive response was defined as a change greater than twofold for CD4^+^AIM^+^ T‐cell responses and a greater than 3‐fold change for CD8^+^AIM^+^ T cell responses. All data were calculated as background subtraction of DMSO‐stimulated values from the spike peptide pool‐stimulated values, with the minimal DMSO level set to 0.005%. Negative responses were excluded from downstream analyses. The limit of quantification (LOQ) was determined by multiplying the geometric mean of all DMSO well values by the geometric standard deviation factor. Samples with background‐subtracted responses exceeding the LOQ were classified as positive responders.

### RNA extraction and real‐time PCR

Total RNA was extracted using TRI reagent (Molecular Research Center, Inc., Cincinnati, OH, USA), and cDNA was synthesised from 1 μg of RNA with random hexamer primers and Moloney murine leukaemia virus reverse transcriptase (Enzynomics, Daejeon, Republic of Korea). Amplification was performed using the TaqMan Universal PCR Master Mix (Applied Biosystems, Foster City, CA, USA) according to the manufacturer's protocol. Briefly, amplification reactions had a total volume of 12 μL and contained 2 μL of cDNA (reverse transcription mixture), oligonucleotide primers (final concentration of 800 nM) and TaqMan hybridisation probe (200 nM). Real‐time PCR probes were labelled at the 5′ end with carboxyfluorescein and at the 3′ end with the quencher carboxytetramethylrhodamine.

To quantify gene expression, cDNA was generated from cellular RNA. Human primers of *ACE2* (Applied Biosystems, Foster City, CA, USA) were used. Real‐time PCR was performed using a QuantStudio (TM) 3 Real‐Time PCR System (Applied Biosystems). The thermocycling parameters were as follows: 95°C for 20 s and then 40 cycles of 95°C for 1 s and 60°C for 20 s. All real‐time PCR data were normalised to the level of glyceraldehyde phosphate dehydrogenase mRNA to correct for variations between samples.

### Immunohistochemistry

A 1 × 1 cm NP tissue sample was collected from a 35‐year‐old male during endoscopic sinus surgery under general anaesthesia, and histologic examination of the NP was performed with haematoxylin and eosin staining.

### Statistical analysis

Assessment of normality was performed using the Shapiro–Wilk and Kolmogorov–Smirnov tests, in combination with considerations of sample size, skewness, and kurtosis. Student's t‐tests or nonparametric tests were used to compare two independent groups (Wilcoxon rank sum test) and two related groups (Wilcoxon signed‐rank test). All *P*‐values are from two‐sided comparisons and descriptive statistics from the compiled flow cytometry data, and statistical analyses were performed using Prism (GraphPad) and R (version 4.5.0, R Foundation for Statistical Computing, Vienna, Austria).

## Author contribution

JW and HC designed the experiments and wrote the study protocols. SMK and SL were responsible for laboratory processing of samples. SJ ran statistical analysis and provided additional material resources. HC and HJK wrote the paper and contributed to the interpretation of the results. SDC and HJK supervised the study.

## Conflict of interest

The authors declare no conflict of interest.

## Supporting information


Supplementary figure 1
Supplementary figure 2
Supplementary figure 3
Supplementary figure 4
Supplementary table 1
Supplementary table 2
Supplementary table 3

Supplementary vedio 1

## Data Availability

The raw sequence data for each sample were deposited in the Gene Expression Omnibus (GEO) under the accession number GSE239595.
