# Supplementary material for: Prolonged persistence of tissue‐resident memory cells in the upper airway following SARS‐CoV‐2 infection and vaccination
Source: Clin Transl Immunology. 2026 Jan 11;15(1):e70075. doi: 10.1002/cti2.70075 (PMC12790937; doi:10.1002/cti2.70075)
Supplement: Supplementary file 1 — Supplementary figure 1 Supplementary figure 2 Supplementary figure 3 Supplementary figure 4 Supplementary table 1 Supplementary table 2 Supplementary table 3 [file CTI2-15-e70075-s001.doc]

**Prolonged persistence of tissue-resident memory cells in the upper airway following SARS-CoV-2 Infection and Vaccination**

**1) Supplementary figures**

**2) Supplementary table**

**Supplementary figure 1.** Comparison of mRNA transcription levels of ACE2 receptors in paired nasopharyngeal (NP) and nasal cavity (NC) mucosa of healthy subjects (N=12).


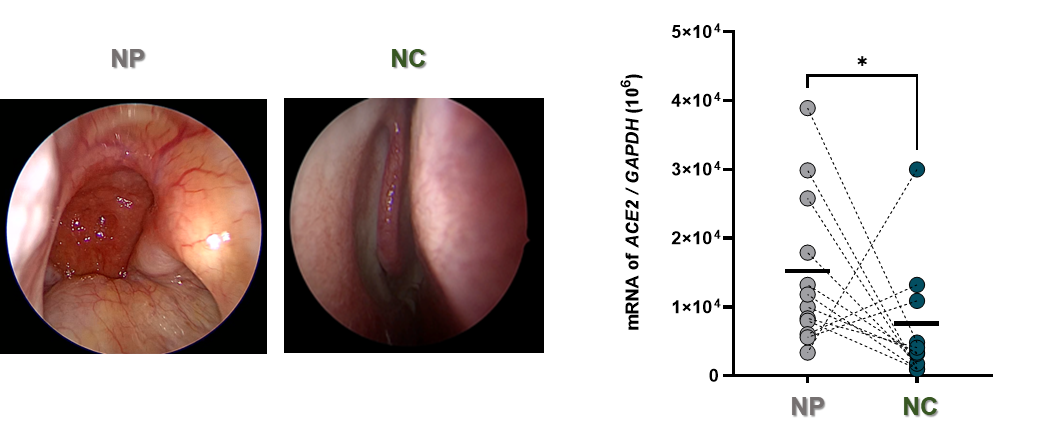


**Supplementary figure 2.** The frequencies of adaptive immune cells in the nasopharyngeal (NP) mucosa of healthy vaccinated and breakthrough subjects.

**(a)** Differences in the frequencies of CD3+ cells as a percentage of CD45+ cells, **(b)** differences in the frequencies of CD4+ T cells as a percentage of CD3+ cells, **(c)** differences in the frequencies of CD8+ T cells as a percentage of CD3+ cells, and **(d)** differences in the frequencies of CD19+ cells as a percentage of CD45+ cells in NP mucosa of healthy vaccinated (HV, N=15) and breakthrough (BR, N=30) subjects (mean + standard deviation: non-parametric t test, *p<0.05).


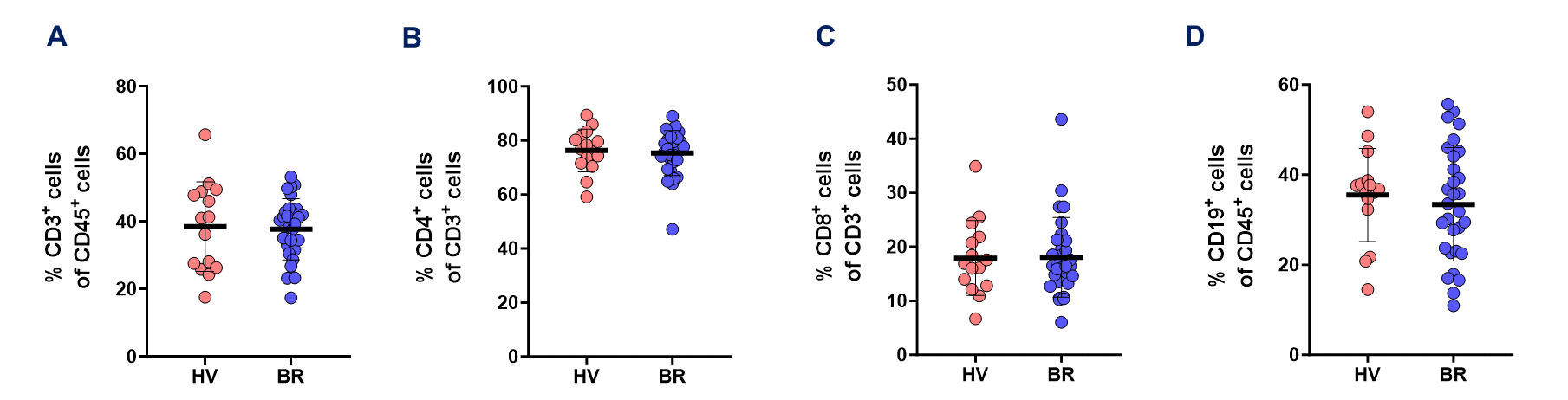


**Supplementary figure 3.** SARS-CoV-2-specific memory CD8+ T cells in the NP mucosa of healthy vaccinated and breakthrough donors.

Differences in subtype profiles of SARS-CoV-2-specific CD4+ memory T cells and ratios in the NPs of the healthy vaccinated (HV, N=15) and breakthrough (BR, N=30) subjects by AIM and ICS assay. **(a)** Representative flow cytometry plots of SARS-CoV-2-specific CD4+ T cells (OX40+ 41BB+) in HV and BR donors and percentage of background-subtracted spike-specific CD4+ T cells (surface OX40+41BB+, as percentage of CD4+ T cells) by AIM assay following 24-hour stimulation of NP immune cells with alpha and omicron spike megapools (MPs). **(b)** Representative flow cytometry plots of SARS-CoV-2-specific IFN-γ+CD4+ T cells (CD40L+IFN-γ+) in the NP of HV and BR donors and percentage of background-subtracted spike-specific CD4+ T cells (surface CD40L+ intracellular IFN-Υ positive, as percentage of CD4+ T cells) by hybrid AIM+ICS following 24-hour stimulation with SARS-CoV-2 alpha and omicron spike MPs for IFN-γ **(b)**. Granzyme B **(c)**, TNF-α **(d)**, and IL-2 **(e)**. **(f)** Donut charts representing the proportion of antigen-specific CD4+ T cells producing 0-4 cytokines in the NP of HV and BR donors (nonparametric t test, *p<0.05, **p<0.01).


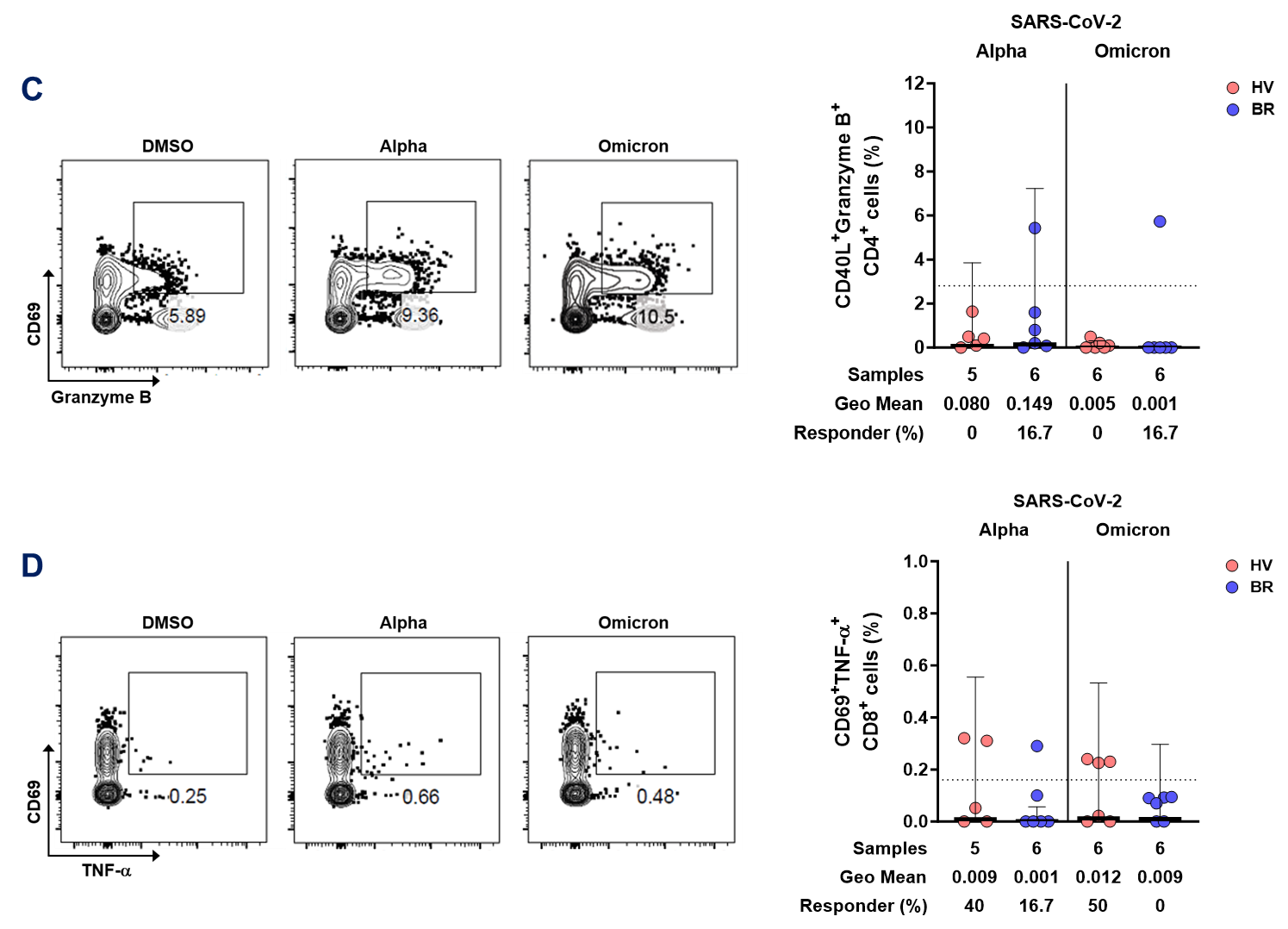

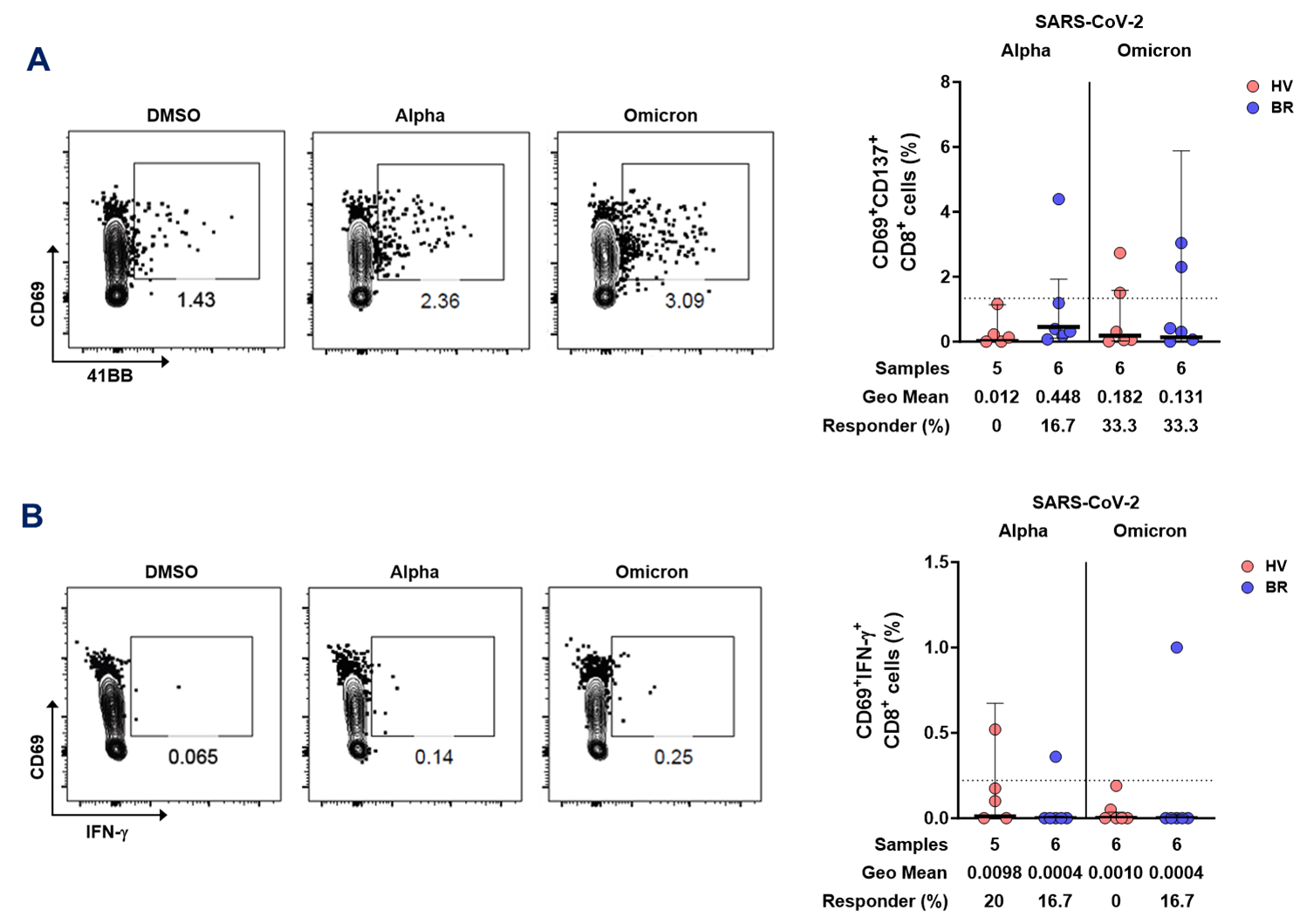


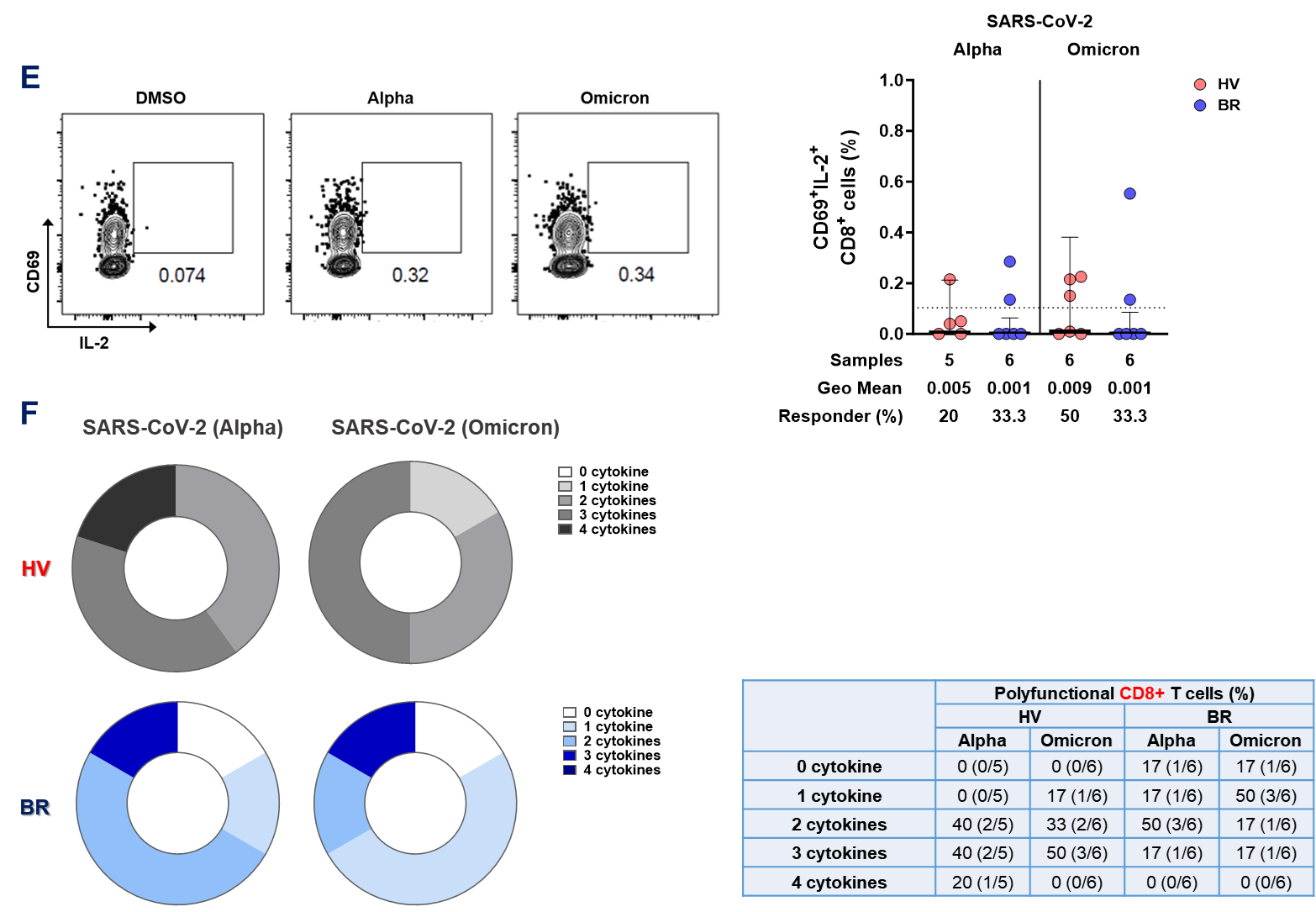


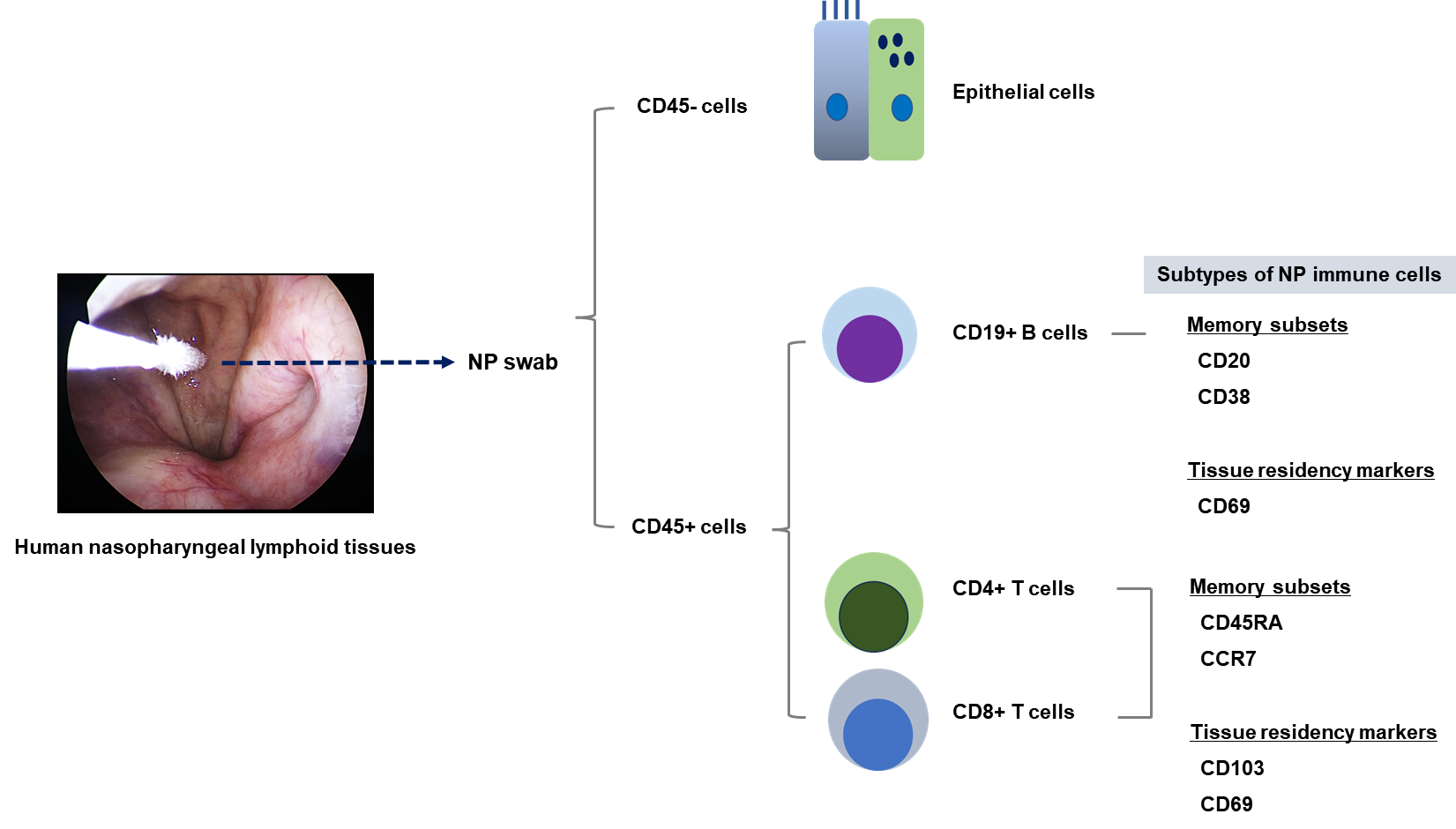
**Supplementary figure 4**. Schematic figure of endoscope-guided nasopharyngeal (NP) brushing from donors and study scheme for the flow cytometry with cellular markers

**Supplementary Table 1. Number of vaccinations and the period between sample collection after the last vaccination in 15 HV subjects**

| **Subjects** | **Number of vaccinations** | **The period since last vaccination (months)** | **The period**  **since last COVID-19 (months)** | **The period between**  **last vaccination and COVID-19 (months)** |
| --- | --- | --- | --- | --- |
| HV1 | 2 | 16 |  |  |
| HV2 | 3 | 22 |  |  |
| HV3 | 3 | 19 |  |  |
| HV4 | 3 | 11 |  |  |
| HV5 | 2 | 28 |  |  |
| HV6 | 3 | 9 |  |  |
| HV7 | 3 | 21 |  |  |
| HV8 | 3 | 32 |  |  |
| HV9 | 3 | 30 |  |  |
| HV10 | 3 | 18 |  |  |
| HV11 | 3 | 29 |  |  |
| HV12 | 2 | 27 |  |  |
| HV13 | 3 | 16 |  |  |
| HV14 | 3 | 15 |  |  |
| HV15 | 3 | 19 |  |  |
| Mean | 2.82 | 20.8 |  |  |

**Supplementary Table 2. Number of vaccinations and the period between sample collection after the last vaccination in 30 BR subjects**

| **Subjects** | **Number of vaccinations** | | **The period since last vaccination (months)** | **The period since last COVID-19 (months)** | **The period between last vaccination and COVID-19 (months)** |
| --- | --- | --- | --- | --- | --- |
| BR1 | | 3 |  | 8 | 7 |
| BR2 | | 3 |  | 22 | 3 |
| BR3 | | 3 |  | 25 | 3 |
| BR4 | | 3 |  | 9 | 6 |
| BR5 | | 2 |  | 20 | 4 |
| BR6 | | 2 |  | 26 | 4 |
| BR7 | | 3 |  | 28 | 8 |
| BR8 | | 3 |  | 21 | 3 |
| BR9 | | 3 |  | 11 | 5 |
| BR10 | | 4 |  | 17 | 8 |
| BR11 | | 3 |  | 10 | 6 |
| BR12 | | 3 |  | 27 | 10 |
| BR13 | | 3 |  | 8 | 9 |
| BR14 | | 3 |  | 15 | 6 |
| BR15 | | 2 |  | 9 | 1 |
| BR16 | | 2 |  | 17 | 7 |
| BR17 | | 3 |  | 28 | 10 |
| BR18 | | 3 |  | 11 | 14 |
| BR19 | | 3 |  | 16 | 6 |
| BR20 | | 3 |  | 21 | 21 |
| BR21 | | 3 |  | 28 | 9 |
| BR22 | | 3 |  | 25 | 23 |
| BR23 | | 3 |  | 7 | 8 |
| BR24 | | 2 |  | 21 | 5 |
| BR25 | | 3 |  | 16 | 15 |
| BR26 | | 3 |  | 26 | 3 |
| BR27 | | 3 |  | 29 | 4 |
| BR28 | | 3 |  | 30 | 17 |
| BR29 | | 3 |  | 14 | 21 |
| BR30 | | 3 |  | 17 | 4 |
| Mean | | 2.86 |  | 18.7 | 8.3 |

**Supplementary Table 3. The list of antibodies for flow cytometry, reagents and software**

| **REAGENT or RESOURCE** | **SOURCE** | | **IDENTIFIER** |
| --- | --- | --- | --- |
| **Antibodies** | |  |  |
| PE/Cy7-conjugated anti CCR7, clone G043H7 | Biolegend | | Cat no: 353225; RRID:AB_11126145 |
| PE/Dazzle594-conjugated anti CD103, Ber-ACT8 | Biolegend | | Cat no: 350223; RRID:AB_2716188 |
| APC/Cy7-conjugated anti CD19, clone HIB19 | Biolegend | | Cat no: 302217; RRID:AB_314247 |
| PE/Cy5-conjugated anti CD20, clone 2H7 | Biolegend | | Cat no: 302307; RRID:AB_314256 |
| FITC-conjugated anti CD3, clone UCHT1 | Biolegend | | Cat no: 300452; RRID:AB_2562046 |
| BV785-conjugated anti CD4, clone OKT4 | Biolegend | | Cat no: 317441; RRID:AB_2563242 |
| AF700-conjugated anti CD45, clone HI30 | Biolegend | | Cat no: 304024; RRID:AB_493760 |
| BV510-conjugated anti CD45RA, clone HI100 | Biolegend | | Cat no: 304141; RRID:AB_2561947 |
| PerCP/Cy5.5-conjugated anti CD324, clone 67A4 | Biolegend | | Cat no: 324113; RRID:AB_2076797 |
| BV605-conjugated anti CD326, clone 9C4 | Biolegend | | Cat no: 324223; RRID:AB_2562518 |
| BV650-conjugated anti CD38, clone HB-7 | Biolegend | | Cat no: 356619; RRID:AB_2566232 |
| BV711-conjugated anti CD69, clone FN50 | Biolegend | | Cat no: 310943; RRID:AB_2566466 |
| APC-conjugated anti CD8, clone SK1 | Biolegend | | Cat no: 344722; RRID:AB_2075388 |
| BV421-conjugated anti CXCR5, clone J252D4 | Biolegend | | Cat no: 356920; RRID:AB_2562302 |
| PE-conjugated anti PD-1, clone EH12.2H7 | Biolegend | | Cat no: 329905; RRID:AB_940481 |
| **Reagent** |  | |  |
| RPMI 1640 | Welgene | | Cat no: LM011-01 |
| Fetal Bovine Serum | ThermoFisher | | Cat no: 16000044 |
| GlutaMAX | ThermoFisher | | Cat no: 35050061 |
| Human BD Fc Block | BD biosciences | | Cat no: 564220 |
| Brilliant Stain Buffer Plus | BD biosciences | | Cat no: 566385 |
| RBC Lysis Buffer | Biolegend | | Cat no: 420301 |
| **Software** |  | |  |
| FlowJo v10.6.2 | BD biosciences | | N/A |
